# Supplementary material for: A Drug Discovery Approach to a Reveal Novel Antioxidant Natural Source: The Case of Chestnut Burr Biomass
Source: Int J Mol Sci. 2024 Feb 21;25(5):2517. doi: 10.3390/ijms25052517 (PMC10931234; doi:10.3390/ijms25052517)
Supplement: Supplementary file 1 [file ijms-25-02517-s001.zip › Supplementary Figures.pdf]

A drug discovery approach to a reveal novel antioxidant natural source: the case of chestnut burr biomass

Alfonso Trezza[1,*,#], Michela Geminiani[1,2,#], Giuseppe Cutrera[1], Elena Dreassi[1], Luisa Frusciante[1], Stefania Lamponi[1,2], Ottavia Spiga[1,2], and Annalisa Santucci[1,2]

[1]Department of Biotechnology Chemistry & Pharmacy; University of Siena, via A. Moro, 53100 Siena, Italy.

[2]SienabioACTIVE, University of Siena, via A. Moro, 53100 Siena, Italy.

*Correspondence: alfonso.trezza2@unisi.it

[#]Both authors equally contributed

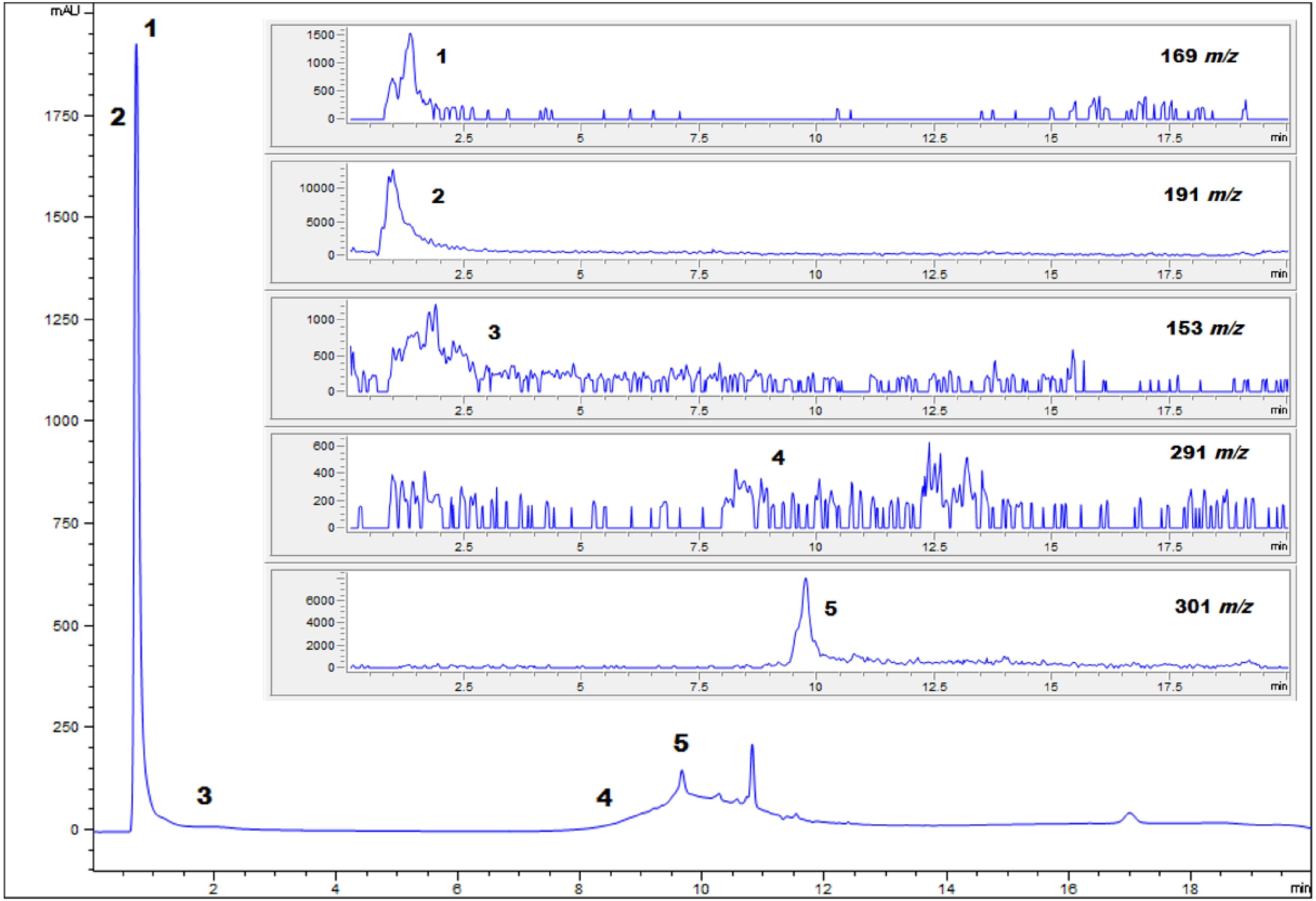

Figure S1. Chromatographic profile of chestnut burrs extract obtained on at 254 nm. Analysis of the chestnut burr extract under the conditions described in the experimental section (in the inset, the contribution of the pseudo-molecular ions of various identified compounds). 1) gallic acid, 2) quinic acid, 3) protocatechuic acid, 4) brevifolin carboxylic acid, and 5) ellagic acid.

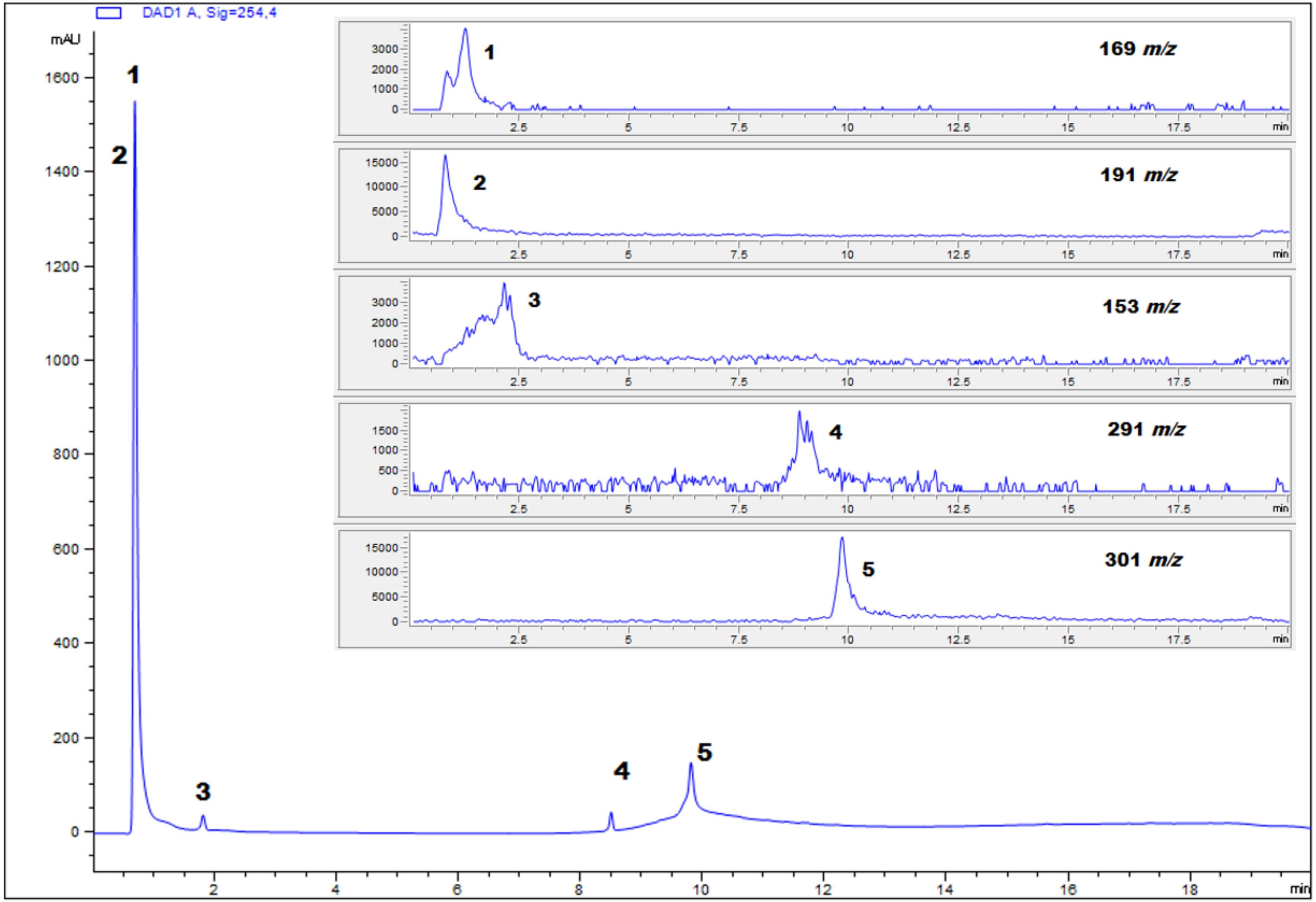

Figure S2. Chromatographic profile of the standard mixture used for the confirmation of the identity obtained at 254 nm. Analysis obtained under the same chromatographic conditions used for the extract (in the inset, the contribution of the pseudo-molecular ions of various standards). 1) gallic acid, 2) quinic acid, 3) protocatechuic acid, 4) brevifolin carboxylic acid, and 5) ellagic acid.

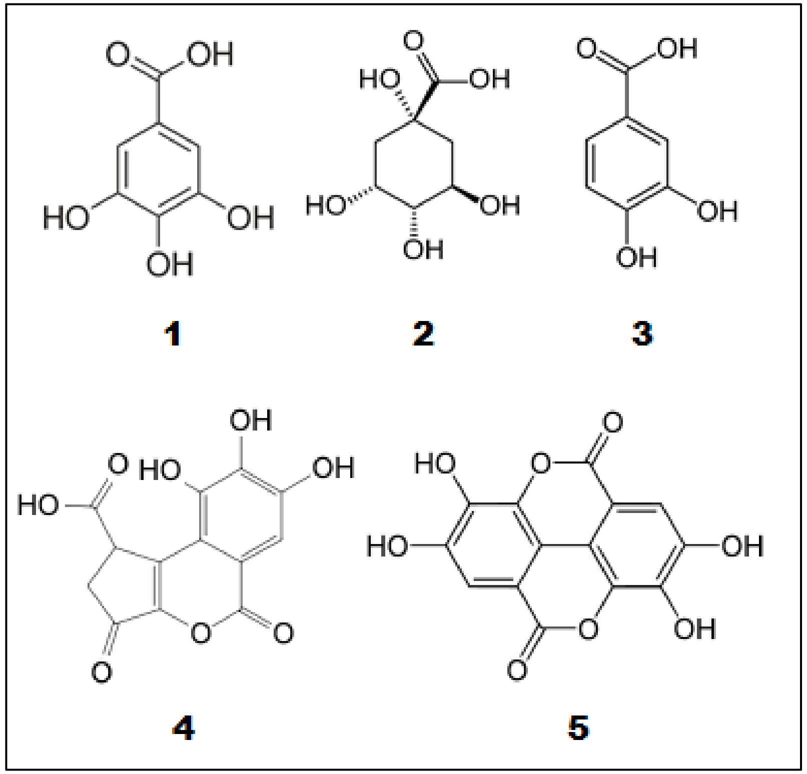

Figure S3. Structures of the species identified in the hydroalcoholic extract of chestnut bur. 1= Gallic acid; 2= Quinic acid; 3= Protocatechuic acid; 4= Brevifolin carboxylic acid; 5= Ellagic acid.

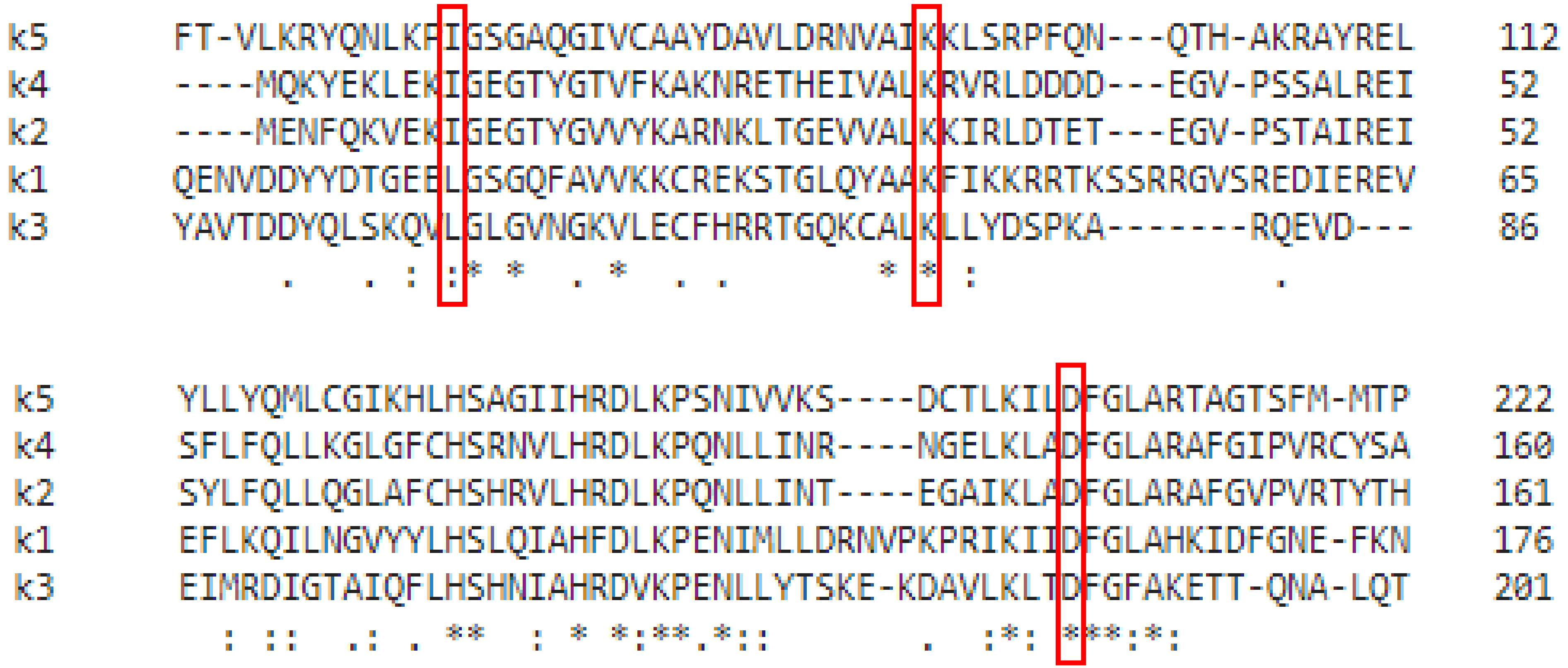

Figure S4. Sequence alignment results between human k1, k2, k3, k4, and k5. Identical and positively conserved amino acids are marked with a star and colon, respectively. Red box highlights all the amino acids involved in hydrogen bond and/or salt bridge with ellagic acid.

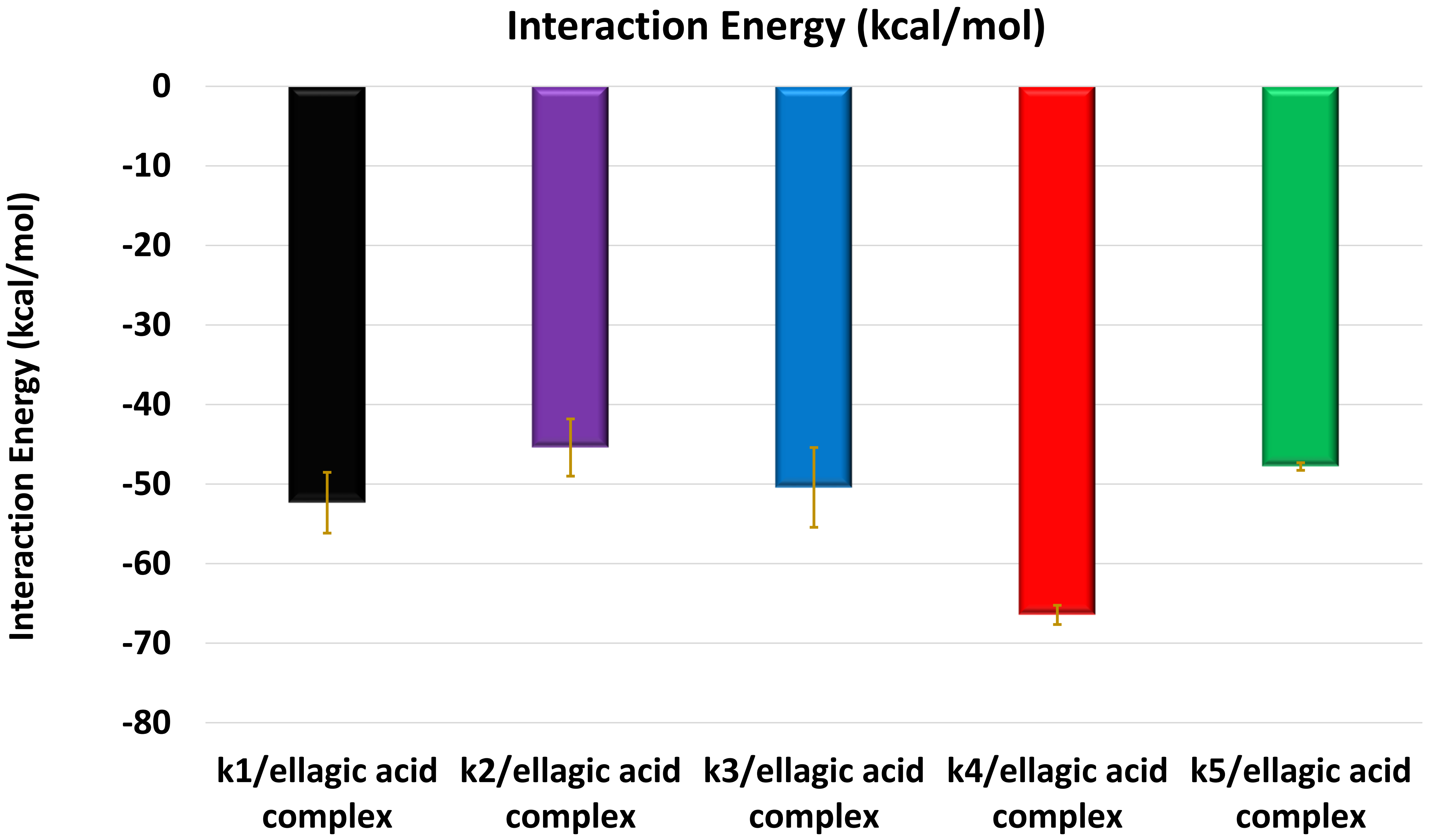

Figure S5. Interaction energy plots. The gold bar reports the error estimation along the entire MD run.

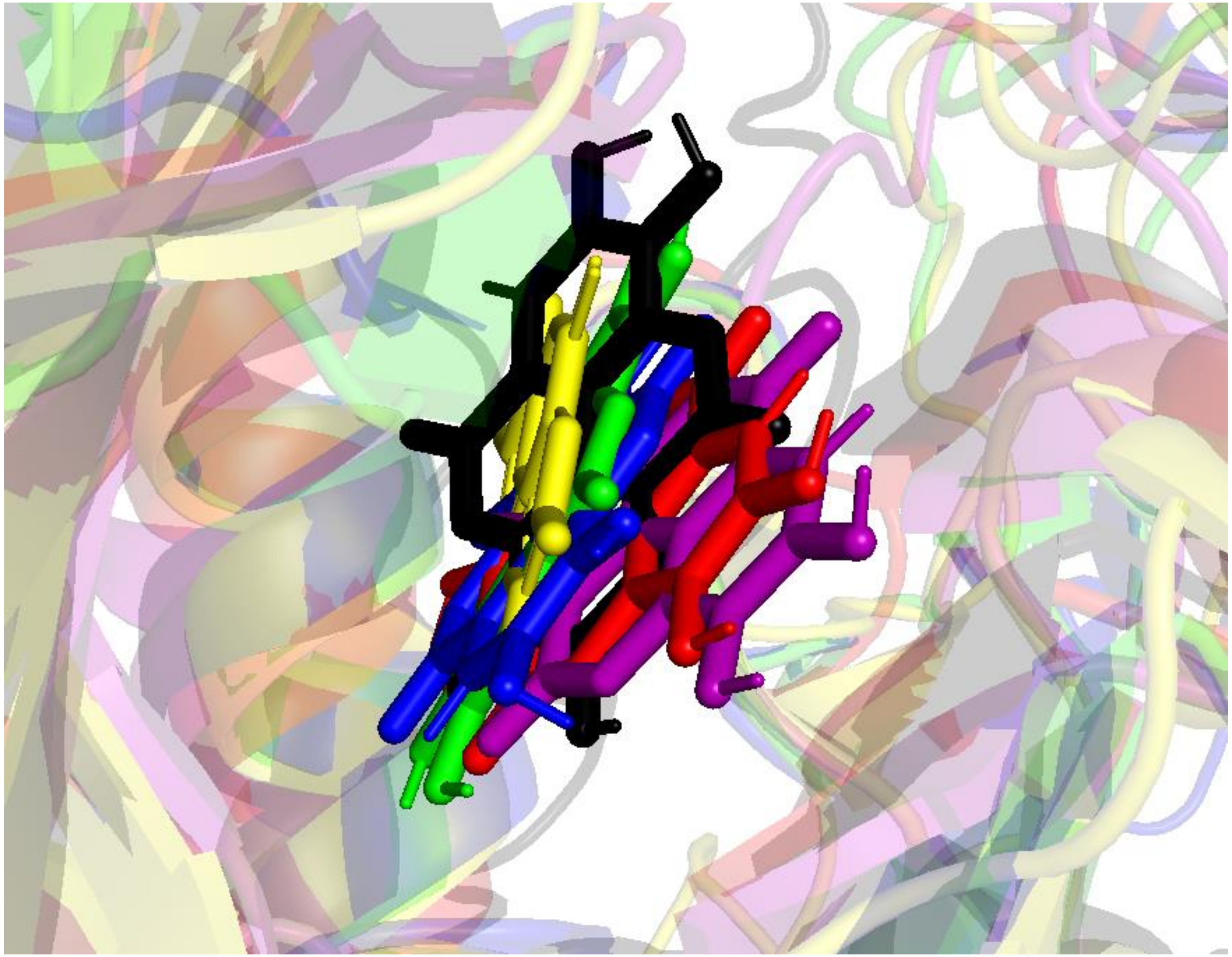

Figure S6. Overview of target/ellagic acid complexes. In transparency cartoon are reported the 3D structures of k1 (green), k2 (purple), k3 (blue), k4 (red), k5 (black), and 2ZJW (yellow) in complex with ellagic acid (in sticks).
