# Supplementary material for: A Drug Discovery Approach to a Reveal Novel Antioxidant Natural Source: The Case of Chestnut Burr Biomass
Source: Int J Mol Sci. 2024 Feb 21;25(5):2517. doi: 10.3390/ijms25052517 (PMC10931234; doi:10.3390/ijms25052517)
Supplement: Supplementary file 1 [file ijms-25-02517-s001.zip › Table S1.pdf]

Protein	Uniprot	Organism	PDB	Method	Resolution (Å)
Deoxyhypusine synthase	P49366	Homo Sapiens	1DHS	X-Ray	2.20
Stress-induced-phosphoprotein 1	P31948	Homo Sapiens	1ELW	X-Ray	1.60
Mitogen-activated protein kinase 14	Q16539	Homo Sapiens	1IAN	X-Ray	2.00
Mitogen-activated protein kinase 10	P53779	Homo Sapiens	1JNK	X-Ray	2.30
Nuclear receptor ROR-alpha	P35398	Homo Sapiens	1N83	X-Ray	1.63
RAC-beta serine/threonine-protein kinase	P31751	Homo Sapiens	1O6L	X-Ray	1.60
Death-associated protein kinase 1	P53355	Homo Sapiens	1P4F	X-Ray	1.90
L-xylulose reductase	Q7Z4W1	Homo Sapiens	1PR9	X-Ray	1.96
Deoxyhypusine synthase	P49366	Homo Sapiens	1RLZ	X-Ray	2.15
Amine oxidase [flavin-containing] B	P27338	Homo Sapiens	1S3E	X-Ray	1.60
Serine/threonine-protein phosphatase 5	P53041	Homo Sapiens	1S95	X-Ray	1.60
Glutaryl-CoA dehydrogenase, mitochondrial	Q92947	Homo Sapiens	1SIQ	X-Ray	2.10
Protein kinase C delta type	Q05655	Homo Sapiens	1YRK	X-Ray	1.70
Amine oxidase [flavin-containing] B	P27338	Homo Sapiens	2BK3	X-Ray	1.80
Amine oxidase [flavin-containing] B	P27338	Homo Sapiens	2C65	X-Ray	1.70
Serine/threonine-protein kinase PAK 4	O96013	Homo Sapiens	2CDZ	X-Ray	2.30
Tyrosine-protein kinase ABL1	P00519	Homo Sapiens	2G1T	X-Ray	1.80
Alpha-ketoglutarate-dependent dioxygenase	Q96Q83	Homo Sapiens	2IUW	X-Ray	1.50
Alpha-aminoadipic semialdehyde dehydrogenase	P49419	Homo Sapiens	2J6L	X-Ray	1.30
Pyruvate dehydrogenase (acetyl-transferring)	Q15118	Homo Sapiens	2Q8G	X-Ray	1.90
5'-AMP-activated protein kinase subunit gamma-1	P54619	Homo Sapiens	2UV4	X-Ray	1.33
Amine oxidase [flavin-containing] B	P27338	Homo Sapiens	2V5Z	X-Ray	1.60
Serine/threonine-protein kinase OSR1	O95747	Homo Sapiens	2VWI	X-Ray	2.15
RAC-beta serine/threonine-protein kinase	P31751	Homo Sapiens	2X39	X-Ray	1.93
Amine oxidase [flavin-containing] B	P27338	Homo Sapiens	2XFN	X-Ray	1.60
Mitogen-activated protein kinase 8	P45983	Homo Sapiens	2XRW	X-Ray	1.33
Amine oxidase [flavin-containing] A	P21397	Homo Sapiens	2Z5X	X-Ray	2.20
Ribosomal protein S6 kinase alpha-1	Q15418	Homo Sapiens	2Z7Q	X-Ray	2.00
Protein kinase C iota type	P41743	Homo Sapiens	3A8X	X-Ray	2.10
Death-associated protein kinase 3	O43293	Homo Sapiens	3BHY	X-Ray	1.24
MAP kinase-activated protein kinase 3	Q16644	Homo Sapiens	3FHR	X-Ray	1.90
MAP kinase-activated protein kinase 3	Q16644	Homo Sapiens	3FXW	X-Ray	2.00

Protein-tyrosine kinase 2-beta	Q14289	Homo Sapiens	3FZS	X-Ray	1.75
Heat shock-related 70 kDa protein 2	P54652	Homo Sapiens	3I33	X-Ray	1.30
Transforming protein RhoA	P61589	Homo Sapiens	3LW8	X-Ray	1.85
Transforming protein RhoA	P61589	Homo Sapiens	3LXR	X-Ray	1.68
ADP-ribosylation factor 6	P62330	Homo Sapiens	3N5C	X-Ray	1.82
Mitogen-activated protein kinase 10	P53779	Homo Sapiens	3OY1	X-Ray	1.70
Tyrosine-protein kinase ABL1	P00519	Homo Sapiens	3PYY	X-Ray	1.85
MAP kinase-activated protein kinase 3	Q16644	Homo Sapiens	3SHE	X-Ray	2.25
NAD-dependent protein deacetylase sirtuin-2	Q8IXJ6	Homo Sapiens	3ZGV	X-Ray	2.27
Histone deacetylase 3	O15379	Homo Sapiens	4A69	X-Ray	2.06
Amine oxidase [flavin-containing] B	P27338	Homo Sapiens	4A7A	X-Ray	1.70
Phosphatidylinositol 4,5-bisphosphate 3-kinase	P48736	Homo Sapiens	4ANV	X-Ray	2.13
Cyclin-dependent-like kinase 5	Q00535	Homo Sapiens	4AU8	X-Ray	1.90
Serine/threonine-protein kinase PAK 4	O96013	Homo Sapiens	4FIH	X-Ray	1.97
Alpha-ketoglutarate-dependent dioxygenase FTO	Q9C0B1	Homo Sapiens	4IE5	X-Ray	1.95
NAD-dependent protein deacetylase sirtuin-1	Q96EB6	Homo Sapiens	4KXQ	X-Ray	1.85
Prolyl 3-hydroxylase OGFOD1	Q8N543	Homo Sapiens	4NHX	X-Ray	2.10
Ribosomal protein S6 kinase alpha-3	P51812	Homo Sapiens	4NW6	X-Ray	1.74
Sphingosine-1-phosphate lyase 1	O95470	Homo Sapiens	4Q6R	X-Ray	2.40
Serine/threonine-protein kinase 24	Q9Y6E0	Homo Sapiens	4QMU	X-Ray	1.55
NAD-dependent protein deacetylase sirtuin-2	Q8IXJ6	Homo Sapiens	4RMH	X-Ray	1.42
Nuclear receptor ROR-alpha	P35398	Homo Sapiens	4S15	X-Ray	1.90
Iodotyrosine deiodinase 1	Q6PHW0	Homo Sapiens	4TTB	X-Ray	2.45
STE20-like serine/threonine-protein kinase	Q9H2G2	Homo Sapiens	4USF	X-Ray	1.75
NAD-dependent protein deacetylase sirtuin-2	Q8IXJ6	Homo Sapiens	4Y6L	X-Ray	1.60
Amyloid-beta precursor protein	P05067	Homo Sapiens	4YN0	X-Ray	2.20
Serine/threonine-protein kinase PAK 1	Q13153	Homo Sapiens	4ZJI	X-Ray	1.99
Transforming protein RhoA	P61586	Homo Sapiens	5A0F	X-Ray	2.00
Death-associated protein kinase 3	O43293	Homo Sapiens	5A6N	X-Ray	1.70
Death-associated protein kinase 1	P53355	Homo Sapiens	5AV4	X-Ray	1.40
TRAF2 and NCK-interacting protein kinase	Q9UKE5	Homo Sapiens	5AX9	X-Ray	2.40
cGMP-dependent protein kinase 2	Q13237	Homo Sapiens	5BV6	X-Ray	1.94
NAD-dependent protein deacetylase sirtuin-2	Q8IXJ6	Homo Sapiens	5D7O	X-Ray	1.63

Ribosomal protein S6 kinase alpha-3	P51812	Homo Sapiens	5D9L	X-Ray	2.15
Serine/threonine-protein kinase 3	Q13188	Homo Sapiens	5DH3	X-Ray	2.47
NAD-dependent protein deacetylase sirtuin-2	Q8IXJ6	Homo Sapiens	5DY4	X-Ray	1.77
Mitogen-activated protein kinase 13	O15264	Homo Sapiens	5EKO	X-Ray	2.00
Protein kinase C theta type	Q04759	Homo Sapiens	5F9E	X-Ray	2.00
Heat shock-related 70 kDa protein 2	P54652	Homo Sapiens	5FPN	X-Ray	1.96
Tyrosine-protein kinase ABL1	P00519	Homo Sapiens	5HU9	X-Ray	1.53
Cyclin-dependent kinase 2	P24941	Homo Sapiens	5K4J	X-Ray	1.60
Proteasome subunit alpha type-7	O14818	Homo Sapiens	5LF4	X-Ray	1.99
Protein kinase C iota type	P41743	Homo Sapiens	5LI9	X-Ray	1.79
ANXA2_HUMAN Annexin A2	P07355	Homo Sapiens	5N7D	X-Ray	2.30
Prolyl hydroxylase EGLN2	Q96KS0	Homo Sapiens	5V1B	X-Ray	2.49
Serine/threonine-protein kinase PAK 4	O96013	Homo Sapiens	5VEF	X-Ray	1.75
Mitogen-activated protein kinase kinase kinase 20	Q9NYL2	Homo Sapiens	5X5O	X-Ray	1.87
Transforming protein RhoA	P61586	Homo Sapiens	6BCB	X-Ray	1.40
cGMP-dependent protein kinase 2	Q13237	Homo Sapiens	6BQ8	X-Ray	2.00
Glucose-6-phosphate 1-dehydrogenase	P53779	Homo Sapiens	6E08	X-Ray	1.90
Mitogen-activated protein kinase 10	P11143	Homo Sapiens	6EMH	X-Ray	1.76
Mitogen-activated protein kinase 10	P53779	Homo Sapiens	6EQ9	X-Ray	1.83
Amine oxidase [flavin-containing] B	P27338	Homo Sapiens	6FW0	X-Ray	1.60
Heat shock protein 105 kDa	Q92598	Homo Sapiens	6GFA	X-Ray	2.00
Mitogen-activated protein kinase 1	P28482	Homo Sapiens	6GJD	X-Ray	1.58
Cyclin-dependent kinase 2	P24941	Homo Sapiens	6GUK	X-Ray	1.30
Cyclin-dependent kinase 2	P24941	Homo Sapiens	6INL	X-Ray	1.75
DNA-(apurinic or apyrimidinic site) endonuclease	P27695	Homo Sapiens	6MKK	X-Ray	1.44
Mitogen-activated protein kinase	Q92918	Homo Sapiens	6NFY	X-Ray	2.17
Deoxyhypusine synthase	P49366	Homo Sapiens	6P4V	X-Ray	1.65
Homeodomain-interacting protein kinase 2	Q9H2X6	Homo Sapiens	6P5S	X-Ray	2.19
DNA-(apurinic or apyrimidinic site) endonuclease	P27695	Homo Sapiens	6P94	X-Ray	2.09
Cyclin-dependent kinase 2	P24941	Homo Sapiens	6Q4K	X-Ray	1.06
Amine oxidase [flavin-containing] B	P27338	Homo Sapiens	6RKP	X-Ray	1.70
Mitogen-activated protein kinase 14	Q16539	Homo Sapiens	6SFI	X-Ray	1.60
Annexin A2	P07355	Homo Sapiens	6TWY	X-Ray	2.30

Deoxyhypusine synthase	P49366	Homo Sapiens	6XXJ	X-Ray	1.41
Deoxyhypusine synthase	P49366	Homo Sapiens	6XXM	X-Ray	1.67
Amine oxidase [flavin-containing] B	P27338	Homo Sapiens	6YT2	X-Ray	1.80
Rho-associated protein kinase 2	O75116	Homo Sapiens	7JNT	X-Ray	2.21

Table S1. List of targets considered in this study.
